# Effects of Clinical Education on Occupational Therapy Students’ Professional Identity: A Cross-Sectional Study in Japan

**DOI:** 10.7759/cureus.79143

**Published:** 2025-02-17

**Authors:** Atsushi Niwa, Yuki Hiraga, Ryuji Matsuda

**Affiliations:** 1 Department of Occupational Therapy, Fukuoka International University of Health and Welfare, Fukuoka, JPN

**Keywords:** clinical education, occupational therapy students, participatory clinical training, personal growth, professional identity

## Abstract

Introduction: Occupational therapy students may form professional identities during clinical training, but this is yet to be verified. This study aimed to clarify the influence of clinical training instructors on the formation of professional identity among occupational therapy students during clinical training. Specifically, we compared the professional identities of occupational therapy students who had not received clinical training with those who had.

Methods: Seventy-five occupational therapy students participated in this cross-sectional study. Of these, 41 were classified into the clinical education group and 34 into the inexperienced group. This study measured professional identity in the clinical education and inexperienced groups. Student’s t-test was conducted for statistical analysis to compare professional identity outcomes in four subcategories: “confidence in personal growth,” “professional pride,” “establishing a view of the medical profession,” and “desire to contribute to society” between the clinical education and inexperienced groups.

Results: When comparing the professional identities of the clinical education and inexperienced groups, significant differences were found in “confidence in personal growth” and “desire to contribute to society” (p > 0.05). However, the factors of “professional pride” and “establishing a view of the medical profession” were not significant (p < 0.05).

Conclusion: The study results showed that the occupational identity dimensions of “confidence in personal growth” and “desire to contribute to society” were significantly higher in the clinical education group than in the inexperienced group.

## Introduction

In recent years, clinical training involving clinical participation has attracted attention, and clinical experience has become more important than traditional training styles [1]. Participatory clinical training is a format in which students participate as part of a medical team and acquire the knowledge, skills, and basic attitudes required by occupational therapists through modeling with a clinical training instructor [2]. 

Theoretical and clinical processes constitute the framework for occupational therapy education, and clinical education has been considered an important part of the traditional occupational therapy curriculum [3]. Occupational therapy clinical education aims to provide occupational therapy students with the professional skills required to apply theoretical concepts to practical situations under the supervision of occupational therapists [4]. The American Occupational Therapy Association has placed clinical education at the core of the occupational therapy curriculum to enable occupational therapy students to integrate knowledge, professional reasoning, and professional practice content and to develop the knowledge, skills, and motivation necessary to become competent occupational therapists [5].

However, in recent years, a decline in motivation to learn among university students has been noted; research has been conducted on the issue of motivation to learn [6]. In psychology, motivation is broadly divided into extrinsic motivation, which avoids shame or punishment for learning (e.g., not being able to earn credit), and intrinsic motivation, which is driven by one’s own interests and curiosity. Intrinsic motivation leads to more effective learning [7]. Identity formation directly promotes independent learning among university students and indirectly through motivation [8]. Furthermore, a study on the relationship between identity as a form of extrinsic motivation and intrinsic motivation reported a positive correlation between the two variables [9]. For these reasons, the effects of occupational therapy clinical training are expected to be felt by students modeling on a professional model (training instructor) during clinical training involving clinical participation. This contributes to the formation of professional identity, leads to intrinsic motivation, and increases motivation to learn; however, these points have not been clarified in detail. Thus, the research question established for this study was whether the professional identity of occupational therapy students is influenced by their clinical training. Specifically, the study aimed to clarify whether clinical education of occupational therapy students at our university influences their professional identity.

## Materials and methods

Study design

This was a cross-sectional study. The participants were occupational therapy students from the Department of Occupational Therapy at Fukuoka International University of Health and Welfare, Faculty of Medical Sciences, Japan. Fukuoka International University of Health and Welfare is an international training institution accredited by the World Federation of Occupational Therapists (WFOT).

Ethical considerations

All patients involved in this study provided informed consent, and the study was approved by the Ethics Review Board of Fukuoka International University of Health and Welfare (approval number: 23-fiuhw-002).

Participants

This study included 34 third- and 41 fourth-year occupational therapy students. The 34 third-year students were assessed before their clinical education, and the 41 fourth-year students were assessed after their second clinical education. Therefore, the third-year occupational therapy students were classified into the inexperienced group (n = 34), and the fourth-year occupational therapy students into the clinical education group (n= 41).

Sample size calculation

In this study, sample size estimation was performed using the G*Power 3 analysis program [10]. The power was set at 0.95, and the significance level (α) was set at 0.01 [11]. In a previous study, the effect size of the clinical education score for occupational therapy was 0.50 [12]. The appropriate sample size for comparing the two groups, the clinical education group and the naive group, was 28 patients per group (n = 56).

Clinical education

The study period for this cross-sectional study was from September 2023 to October 2024. The students attended university lectures according to the curriculum of the Department of Occupational Therapy from the third to the final year and from the first to the final year of university for four years (university curriculum).

According to the curriculum of Fukuoka International University of Health and Welfare or the Department of Occupational Therapy, occupational therapy students are required to attend lectures from the first to fourth years [12]. The curriculum gradually progresses from basic fields in the first year to specialized fields tailored to occupational therapy evaluation in the second year. In the third year, students receive clinical education based on occupational therapy practice at medical institutions [12].

In the occupational therapy evaluation training, students are assigned to actual patients in hospitals or facilities across areas such as physical, mental, developmental, and geriatric disabilities. They conduct occupational therapy evaluations and then consider the necessary occupational therapy practice over 20 days [12]. In the comprehensive occupational therapy training, students are assigned to patients in hospitals and facilities in the same areas and gain experience in assessment, planning, and implementation of occupational therapy over 80 days. This training equips them with the knowledge, skills, and motivation required to become occupational therapists [12]. The clinical education provided to the students in the present study comprised both occupational therapy assessment and comprehensive occupational therapy training [12].

Assessment

The clinical education group (n = 41) and the inexperienced group (n = 34) were administered measures of professional identity.

Professional identity　

Professional identity was assessed using survey items on professional identity among medical students conducted by Iwata et al. [13]. The participants were asked to respond to 32 questions on a five-point scale from 1 “not at all” to 5 “a great deal.” The 32 questions were classified into four subcategories: “confidence in personal growth,” “professional pride,” “establishing a view of the medical profession,” and “desire to contribute to society” [13]. A higher score on the professional identity scale indicated a more established professional identity.

Statistical analysis

The Shapiro-Wilk test was conducted for the distribution of normality. Student’s t-test was conducted to analyze age differences, while chi-square tests were conducted to examine sex disparities, both at a significance level of 5%. Student’s t-test was also performed with a risk level of 5% to compare the interpretations of professional identity, namely, “confidence in personal growth,” “professional pride,” “establishing a view of the medical profession,” and “desire to contribute to society,” before and after the clinical education among the occupational therapy students. The effect size indicators and standards were set as follows: small: 0.20; medium: 0.50; and large: 0.80. Statistical analyses were performed using JMP version 14.0 (JMP, Cary, USA).

## Results

Participant characteristics

Table 1 presents the participants’ basic attributes. The participants were 75 occupational therapy students who were divided into two groups: the clinical education group (n = 41) and the inexperienced group (n = 34). The results of the baseline analysis indicated no significant differences in sex or age between the two groups.

**Table 1:** Participant characteristics Values are expressed as means ± standard deviation Age differences were assessed by conducting Student’s t-test, while sex disparities were assessed using chi-square tests.

	Clinical education group (n = 41)	Inexperienced group (n = 34)	t or χ^2^	p
Age (years)	21.1 ± 0.1	20.7 ± 0.9	3.96	0.135
Male, n (%)	9 (22.0)	13 (38.2)	2.37	0.126
Female, n (%)	32 (78.0)	21 (61.8)		

Analysis of the professional identity results

**Figure 1:**
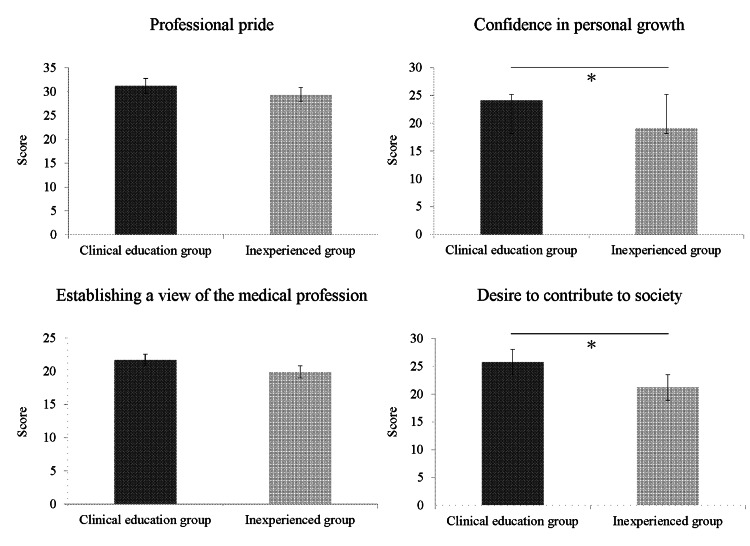
Comparison of the clinical education group and the inexperienced group * Significant differences were observed between the clinical education group and the inexperienced group (p < 0.01)

Table 2 summarizes the clinical education and inexperienced groups’ outcomes. A comparison between the clinical education group and the inexperienced group is also shown in Figure 1. Differences in professional identity between the two groups were analyzed by conducting Student’s t-test, revealing significant results for “confidence in personal growth” and “desire to contribute to society” (all p < 0.01) for both groups. These results suggest that receiving approximately 100 days of clinical education from occupational therapist instructors promoted the students’ “confidence in personal growth” and “desire to contribute to society.” However, as we could not analyze the specific progression of clinical education for each student, the exact process leading to these developments remains unclear.

**Table 2:** Results of the outcome measures Values are expressed as means ± standard deviation CI; confidence interval. Effect size: The effect size standard was small (0.20), medium (0.50), and large (0.80)

	Clinical education group (n=41)	Inexperienced group (n=34)	t	p	Mean (95% CI)	Effect size
Professional pride	31.2 ± 8.7	29.4 ± 9.2	0.86	0.39	1.8 (-2.3 to 5.9)	0.20
Confidence in personal growth	24.1 ± 5.7	19.1 ± 5.9	3.70	0.01	4.9 (2.03 to 7.6)	0.88
Establishing a view of the medical profession	21.6 ± 4.1	19.8 ± 6.1	1.52	0.13	1.8 (-0.5 to 4.1)	0.36
Desire to contribute to society	25.7 ± 6.3	21.2 ± 7.1	2.92	0.01	4.5 (1.4 to 7.6)	0.68

In contrast, “professional pride” and “establishing a view of the medical profession” did not differ significantly between the two groups (all p > 0.05). These results suggest that even with the approximately 100 days of clinical education under the guidance of the occupational therapists, the students were not affected in terms of “professional pride” or “establishing a view of the medical profession.” Given the scarcity of an occupational therapy system in Japan that accounts for the country’s cultural and social backgrounds, ensuring “professional pride” and “establishing a view of the medical profession” may be difficult.

Furthermore, the effect size for “professional pride” was small at 0.20, for “confidence in personal growth”, it was large at 0.88, for “establishing a view of the medical profession”, it was small at 0.36, and for “desire to contribute to society” was medium at 0.68. These results suggest that the impact of clinical education on professional integrity primarily stems from its effects on “confidence in personal growth” and “desire to contribute to society.” However, based on the effect sizes, the effect of clinical education on “professional pride” and “establishing a view of the medical profession” appears to be limited.

In addition, the differences in each outcome between the clinical education group and the inexperienced group were as follows: 1.8 (-2.3 to 5.9) for “professional pride,” 4.9 (2.0 to 7.6) for “confidence in personal growth,” 1.8 (-0.5 to 4.1) for “establishing a view of the medical profession,” and 4.5 (1.4 to 7.6) for “desire to contribute to society.” The differences for “professional pride” and “establishing a view of the medical profession” were approximately 1.8, while the differences for “confidence in personal growth” and “desire to contribute to society” were approximately 5, highlighting a numerical disparity. However, since minimal clinically important differences have not been reported for measures of professional identity [13], the clinical significance of these differences remains unclear.

## Discussion

This study aimed to clarify the influence of clinical training instructors on the formation of professional identity among occupational therapy students during clinical education in our department. To this end, participants were classified into two groups, namely, the clinical education group and the inexperienced group, and their professional identities were compared. The results showed that the occupational identity dimensions of “confidence in personal growth” and “desire to contribute to society” were significantly higher in the clinical education group than in the inexperienced group. However, “professional pride” and “establishing a view of the medical profession” did not reach significance. Therefore, by receiving clinical education from clinical training instructors, occupational therapy students can develop a professional identity, such as “confidence in personal growth” and “desire to contribute to society,” which is a novel aspect of this study.

Previous research has shown that students’ professional identities are formed through active communication with faculty [14]. Furthermore, professional identity formation includes entering into clinical roles, enriching clinical work, and personal growth [15]. Therefore, we believe that the clinical education provided in this study was thorough and students were given proactive roles, which may explain why “confidence in personal growth” and “desire to contribute to society” showed higher values ​​in the clinical education group.

However, “professional pride” and “establishing a view of the medical profession” did not reach significance in the clinical education or the inexperienced group. Previous studies have reported that time spent in professional roles also influences professional identity [16]. The duration of the clinical education in this study was 100 days, with some participants receiving 20 and others receiving 80 days. It is possible that professional identity such as “professional pride” and “establishing a view of the medical profession” was not formed. Occupational therapists’ professional identity formation involves occupation-centered practice, ontological reflexivity, theory-practice alignment, and professional socialization [17]. Professional pride and social contribution associated with this identity require long-term practice, both as a student and as a professional [18]. Therefore, we believe that gaining extensive practical experience in one’s specialty is essential for developing a professional identity characterized by “professional pride” and for “establishing a view of the medical profession.” In other words, in this study, the clinical education group also showed high scores for the professional identity items “confidence in personal growth” and “desire to contribute to society,” while no significance was observed in either the clinical education group or the inexperienced group for “professional pride” and “establishing a view of the medical profession.” Therefore, although the clinical education conducted in this study did not lead to the formation of “professional pride” and “establishing a view of the medical profession,” it may be possible to encourage the formation of “confidence in personal growth” and “desire to contribute to society.” Based on these findings, enhancing clinical education and university education is necessary to promote the formation of professional identity in occupational therapy education [19].

Regarding the clinical usefulness of the findings, we believe that incorporating approximately 100 days of clinical education into the university curriculum can help foster a professional identity rooted in “confidence in personal growth” and a “desire to contribute to society.” Furthermore, by working long-term as occupational therapists, individuals can develop “professional pride” and “establish a view of the medical profession.” To achieve this, establishing a system that ensures a consistent connection between university education and post-graduate education (career development) is necessary.

Although this study offers valuable insights, it also has some limitations. First, the study was cross-sectional and conducted at a single university, which makes it difficult to generalize the findings. Second, the participants were divided into a clinical education group and a non-clinical education group based on their clinical education, so inferring a causal relationship between the two groups is not possible. Third, the clinical education group received clinical education in hospitals, elderly care facilities, developmental support centers, and psychiatric rehabilitation facilities, but the specific characteristics of each facility were not examined thoroughly. Consequently, we could not analyze in detail how “confidence in personal growth” and “desire to contribute to society” evolved during the clinical education process, while “professional pride” and “establishing a view of the medical profession” did not. Fourth, the content of the clinical education likely varied between institutions, making it challenging to explore all aspects in detail. To address this situation, it may be possible to first select a training field that corresponds to the students’ interests. Thus, in the future, a more detailed analysis could be possible by investigating the specific training fields that students are interested in. Finally, the study only considered age and sex as participant attributes, disregarding other potential confounding factors such as work experience, personality traits, and motivation. Future studies should consider these limitations. 

## Conclusions

This study aimed to clarify whether the clinical education of occupational therapy students at our university influences their professional identity. The results showed that the occupational identity dimensions of “confidence in personal growth” and “desire to contribute to society” were significantly higher in the clinical education group than in the inexperienced group. However, “professional pride” and “establishing a view of the medical profession” did not reach significance. These results suggest that clinical education can promote the formation of professional identities among occupational therapy students.

## Appendices

**Table 3:** Exploring the occupational therapy students’ professional identity Subitem (Question number) Professional pride (9, 13,16, 21, 25, 26, 30, 31) Confidence in personal growth (1, 2, 6, 8, 10, 14, 17, 18, 19, 23) Establishing a view of the medical profession (3, 5, 7, 11, 12, 15) Desire to contribute to society (4, 20, 22, 23, 24, 27, 28, 29, 32) The professional identity scale developed by Iwata et al. [13] was translated by the first author, Atsushi Niwa for use in this study, with permission from the original authors.

1	I feel that being an occupational therapist suits me
2	I am glad that I chose to be an occupational therapist
3	I want to contribute to the world of occupational therapy as an occupational therapist
4	I feel that I can always work in my own way
5	I want to respond to patients’ wishes as an occupational therapist
6	I want to continue working as an occupational therapist for the rest of my life
7	I want to help patients as an occupational therapist
8	I am proud to be an occupational therapist
9	I think I can become a therapist that patients need
10	I cannot imagine doing anything other than being an occupational therapist
11	I want to contribute to society as an occupational therapist
12	I want to produce unique results as an occupational therapist that others cannot
13	I think I can become indispensable in the medical world
14	I feel I can continue to grow as an aspiring occupational therapist
15	I want to contribute to the development of medicine as an occupational therapist
16	I think I can have a unique academic background
17	I am proud to tell others that I am a student who aspires to be an occupational therapist
18	I can grow as a person through my work as an occupational therapist
19	I think I will be able to live my own life as an occupational therapist in the real world
20	I have my own ideas about what occupational therapy should be
21	I think I can support patients
22	I think I will be able to fully realize my potential in the real world
23	I think becoming an occupational therapist is the way I want to live my life
24	I want to be able to demonstrate my individuality in my relationships with doctors as an occupational therapist
25	I feel like I have become my own person as an occupational therapist
26	I think I can become a therapist that many people will continue to need
27	I am clear about what kind of occupational therapist I want to be
28	I think I will be able to become a unique occupational therapist in the future
29	I am clear about what kind of occupational therapy I want to provide
30	As a member of a medical team, I think I can become a therapist who will be increasingly needed in the future
31	I think I can become a therapist who can make unique contributions as a member of a medical team
32	I think I can provide unique occupational therapy
